# Dissemination of Multidrug-Resistant Bacteria into the Arctic

**DOI:** 10.3201/eid1401.070704

**Published:** 2008-01

**Authors:** Maria Sjölund, Jonas Bonnedahl, Jorge Hernandez, Stina Bengtsson, Gunilla Cederbrant, Jarone Pinhassi, Gunnar Kahlmeter, Björn Olsen

**Affiliations:** * Central Hospital, Växjö, Sweden; † Kalmar County Hospital, Kalmar, Sweden; ‡ Kalmar University, Kalmar, Sweden; § Uppsala University, Uppsala, Sweden; 1 Current affiliation: Centers for Disease Control and Prevention, Atlanta, Georgia, USA

**Keywords:** Escherichia coli, antimicrobial drug resistance, Arctic birds, dispatch

## Abstract

We show that *Escherichia coli* isolates originating from Arctic birds carry antimicrobial drug resistance determinants. This finding implies that dissemination of drug-resistant bacteria is worldwide. Resistance genes can be found even in a region where no selection pressure for resistance development exists.

Bacteria display a unique ability to adapt to changes in their environment and to develop mechanisms to protect themselves against toxic compounds. Their ability to develop resistance mechanisms to antimicrobial drugs has assumed catastrophic proportions, rendering more and more infections difficult or impossible to treat (*1*). Most reports suggest that the main force behind emergence of drug resistance is the use and misuse of antimicrobial drugs during the past few decades, but there is also evidence for the epidemic spread of drug-resistant bacteria as a contributing factor (*2*).

## The Study

We investigated bacteria from a region considered to be one of the last outposts of wilderness, the Arctic, with the belief that in this region human influence on the ecology of antimicrobial resistance would be minimal. Antimicrobial drug resistance in *Escherichia coli* isolated on site from fecal or cloacal swabs of Arctic birds was studied.

During the Beringia expedition organized by the Swedish Polar Research Secretariat in 2005, fecal or cloacal samples were collected from 97 birds in 3 geographic regions: northeastern Siberia; Point Barrow, Alaska, USA (71º30′N, 156º78′W); and northern Greenland. Samples were transported in a swab-transport system to a laboratory on the expedition ship. Upon arrival, the samples were immediately cultured, and single colonies of enterobacteria were isolated on blood agar plates (*3*). One isolate of *E*. *coli* was selected from each sample and stored at –70°C for further analysis. Susceptibility to 17 antimicrobial drugs was determined by using disk diffusion with disks on Iso-Sensitest agar (Oxoid, Basingstoke, UK) in accordance with the recommendations of the Swedish Reference Group for Antibiotics (*4*). MIC determinations were performed by using the E-test (AB Biodisk, Solna, Sweden) on Iso-Sensitest agar. All bacteria were tested against the following antimicrobial drugs: ampicillin, cefadroxil, cefuroxime, cefpodoxime (to screen for *E*. *coli* with extended-spectrum β-lactamases), chloramphenicol, ciprofloxacin, fosfomycin-trometamol, gentamicin, imipenem, mecillinam, nalidixic acid, nitrofurantoin, streptomycin, sulfamethoxazole, tetracycline, trimethoprim, and tigecycline.

Clonal diversity of the 97 isolates was assessed by using a commercial typing system, the PhenePlate (PhP) system (PhPlate Microplate Techniques AB, Stockholm, Sweden). This system uses results of 11 biochemical reactions in a microplate to obtain a biochemical fingerprint of each tested isolate and has been used for typing of *E*. *coli* (*5**,**6*). Compared with traditional qualitative biochemical testing where the results are read once, PhP typing is based on several readings and thereby includes dynamics of the reaction in the analysis. PhP typing was performed as described with 2 modifications: 1 of the 11 reagents, melabionate, was replaced with raffinose, and *E*. *coli* reference strain ATCC 25922 was used as a positive control (*6*). Biochemical fingerprints obtained were compared with each other, and similarity was calculated by using PhPWIN4 software (*6*). PhP types with >2 isolates with the same biochemical fingerprint were designated as common types (CTs); types with 1 isolate, which represented rare or unique clones, were designated as single (Si) types.

*E*. *coli* isolates from Arctic birds carried antimicrobial drug resistance determinants; among 17 antimicrobial drugs tested, resistance to 14 was detected. Resistance was observed in 8 isolates, 4 of which displayed resistance to >4 drugs (Table), and occurred most often to ampicillin, sulfamethoxazole, trimethoprim, chloramphenicol, and tetracycline. Two resistant isolates displayed isolated fosfomycin resistance with MIC values of 256 mg/L and 1,024 mg/L. No resistance to gentamicin, imipenem, or tigecycline was observed.

**Table:** Antimicrobial drug resistance phenotypes in 8 *Escherichia coli* isolates from Arctic birds

Isolate	Avian host species	Geographic origin	PhenePlate type*	Drug resistance profile†
2	Western sandpiper	Lorino, Siberia	Si	Amp, Cpd, Cdr, Cxm
18	Vega/Glaucous gull	Novo Chaplino, Siberia	CT3	Amp, Cpd
26	Vega/Glaucous gull	Novo Chaplino, Siberia	Si	Amp, Sul, Chl, Tet, Tri, Nit, Str
35	Vega/Glaucous gull	Novo Chaplino, Siberia	Si	Fos
36	Vega/Glaucous gull	Novo Chaplino, Siberia	Si	Fos
75	Emperor/Brent goose	Kolyuchin, Siberia	CT5	Tet
94	Iceland/Glaucous gull	Thule, Greenland	CT5	Amp, Sul, Chl,Mec, Tet, Tri, Str
97	Iceland/Glaucous gull	Thule, Greenland	Si	Amp, Sul, Chl, Mec, Tri, Str, Nal, Cip

*Si, single type; CT, common type. †Amp, ampicillin; Cpd, cefpodoxime; Cdr, cefadroxil; Cxm, cefuroxime; Sul, sulfamethoxazole; Chl, chloramphenicol; Tet, tetracycline; Tri, trimethoprim; Nit, nitrofurantoin; Str, streptomycin; Fos, fosfomycin; Mec, mecillinam; Nal, nalidixic acid; Cip, ciprofloxacin.

PhP typing divided the 97 isolates into 11 CTs (CT1–CT11) and 34 Si types (Appendix Table). The 3 most frequent CTs were CT3, CT2, and CT5, which contained 29, 9, and 5 isolates, respectively. Among the isolates from Lorino, Siberia (65º39′N, 172º15′W), (n = 13), Si types were most common (n = 5). Likewise, among isolates from Novo Chaplino, Siberia (64º70′N, 173º00′W) (n = 33), Si types (n = 19) predominated. In contrast, only 2 Si types were found among isolates from Kolyuchin, Siberia (67º04′N, 173º21′W) (n = 29). Most of these isolates (n = 18) were defined as CT3. Six of the 11 CTs were restricted to 1 sample site: CT1 at Lorino, CT6 at Novo Chaplino, CT7 at Kolyuchin, CT9 and CT10 at Wrangel Island (70º55′N, 179º29′W), and CT11 at Thule, Greenland (76º32′N, 68º44′W). Among the 8 drug-resistant isolates, 1 was CT3 and 2 were CT5; the other 5 were defined as Si types (Table).

## Conclusions

Antimicrobial drug resistance in *E*. *coli* isolated from wild birds has been described (*7**,**8*). A high frequency of *E*. *coli* isolates from migratory Canada geese sampled on the eastern shore of Maryland in the United States were resistant to penicillin G, ampicillin, cephalothin, and sulfathiazole (*8*). Similarly, a high prevalence of antimicrobial drug–resistant *E*. *coli* isolated from black-headed gulls in the Czech Republic has been observed (*7*).

There are several explanations for antimicrobial drug resistance in the normal microbiota of Arctic birds. First, resistance may develop de novo through spontaneous mutation(s) (*9*). Second, resistance can be acquired by horizontal gene transfer from other microbes; many bacteria and fungi constitute natural sources of drug resistance genes and may serve as reservoirs in the environment (*10*). Third, bacteria with antimicrobial drug resistance could be imported into the region either by migratory birds or through human refuse (food, excretions) from fishermen, settlers, and prospectors in the area. The region around the Bering Strait constitutes the breeding ground of a large number of waterfowl, geese, and shorebirds. Although many of these species are largely confined to one side of the Bering Strait, other species pass through both continents during migration or occasionally wander across the strait (*11*). Moreover, bird species that spend the winter in up to 6 different continents can be found in this area (*11*). Thus, migratory birds that have acquired drug-resistant bacteria during wintering or stops at lower latitudes before migrating to the Arctic provide a potential explanation for introduction of drug resistance into this region. The fact that 1 isolate from a juvenile Western sandpiper sampled far from human settlements on the tundra had resistance to cefadroxil, cefuroxime, and cefpodoxime, a resistance pattern commonly seen in clinical isolates, supports the theory of introduction by migration and transfer of bacteria between birds.

To the best of our knowledge, the most remote and isolated environment investigated for drug-resistant bacteria is the Bolivian community of 130 Guaraní Indians, as described by Bartoloni et al. (*12*). This community is located at an altitude of ≈1,700 m and can only be reached by a 3-hour steep climb. Nevertheless, high carriage rates of drug-resistant commensal *E*. *coli* were found in this community, although exposure to antimicrobial drugs in the area had been limited. Similarly, antimicrobial drug–resistant bacteria in wild animals with little or no contact with human settings has been reported (*13**,**14*). These findings and those of our study suggest that commensal bacteria in humans and animals constitute hidden reservoirs of antimicrobial drug resistance (*15*). A possible explanation for the unexpectedly high carriage rate of drug-resistant *E*. *coli* in the Indian community in Bolivia is the importation of drug-resistant isolates by migratory birds.

We have shown that antimicrobial drug resistance genes are present in 1 of the most remote areas on Earth, the Arctic. Resistant as well as multiresistant isolates of *E*. *coli* were detected in the normal flora of Arctic birds. This finding highlights the unique nature of bacterial adaptation and the complexity of dissemination of antimicrobial drug resistance. To fully understand the extent of environmental and commensal reservoirs of resistance, studies of antimicrobial drug resistance in different habitats are warranted.

## Supplementary Material

Appendix Table Origin and PhenePlate types of 97 Escherichia coli isolates from Arctic birds
