# Supplementary material for: Dissemination of Multidrug-Resistant Bacteria into the Arctic
Source: Emerg Infect Dis. 2008 Jan;14(1):70–2. doi: 10.3201/eid1401.070704 (PMC2600168; doi:10.3201/eid1401.070704)
Supplement: Appendix Table — Origin and PhenePlate types of 97 Escherichia coli isolates from Arctic birds [file 07-0704_appT-s1.pdf]

Appendix Table. Origin and PhenePlate types of 97 *Escherichia coli* isolates from Arctic birds

Isolate no.	Avian host species*	Geographic origin	PhenePlate type†
1	Western sandpiper	Lorino, Siberia	CT1
2	Western sandpiper	Lorino, Siberia	Si
3	Dunlin	Lorino, Siberia	Si
4	Western sandpiper	Lorino, Siberia	CT2
5	Red-necked stint	Lorino, Siberia	CT1
6	Dunlin	Lorino, Siberia	Si
7	Red-necked stint	Lorino, Siberia	CT3
8	Red-necked stint	Lorino, Siberia	CT3
9	Red-necked stint	Lorino, Siberia	CT3
10	Dunlin	Lorino, Siberia	Si
11	Red-necked stint	Lorino, Siberia	Si
12	Yellow wagtail	Lorino, Siberia	CT4
13	Red-necked stint	Lorino, Siberia	CT3
14	Vega/Glaucus gull	Novo Chaplino, Siberia	Si
15	Kittiwake	Novo Chaplino, Siberia	Si
16	Vega/Glaucus gull	Novo Chaplino, Siberia	CT2
17	Vega/Glaucus gull	Novo Chaplino, Siberia	Si
18	Vega/Glaucus gull	Novo Chaplino, Siberia	CT3
19	Vega/Glaucus gull	Novo Chaplino, Siberia	CT5
20	Vega/Glaucus gull	Novo Chaplino, Siberia	CT3
21	Vega/Glaucus gull	Novo Chaplino, Siberia	Si
22	Vega/Glaucus gull	Novo Chaplino, Siberia	Si
23	Vega/Glaucus gull	Novo Chaplino, Siberia	Si
24	Vega/Glaucus gull	Novo Chaplino, Siberia	Si
25	Vega/Glaucus gull	Novo Chaplino, Siberia	Si
26	Vega/Glaucus gull	Novo Chaplino, Siberia	Si
27	Vega/Glaucus gull	Novo Chaplino, Siberia	CT3
28	Vega/Glaucus gull	Novo Chaplino, Siberia	Si
29	Vega/Glaucus gull	Novo Chaplino, Siberia	CT2
30	Vega/Glaucus gull	Novo Chaplino, Siberia	Si
31	Vega/Glaucus gull	Novo Chaplino, Siberia	Si
32	Vega/Glaucus gull	Novo Chaplino, Siberia	Si
33	Vega/Glaucus gull	Novo Chaplino, Siberia	Si
34	Vega/Glaucus gull	Novo Chaplino, Siberia	Si
35	Vega/Glaucus gull	Novo Chaplino, Siberia	Si
36	Vega/Glaucus gull	Novo Chaplino, Siberia	Si
37	Vega/Glaucus gull	Novo Chaplino, Siberia	CT5
38	Vega/Glaucus gull	Novo Chaplino, Siberia	CT4
39	Vega/Glaucus gull	Novo Chaplino, Siberia	Si
40	Vega/Glaucus gull	Novo Chaplino, Siberia	CT5
41	Vega/Glaucus gull	Novo Chaplino, Siberia	CT6
42	Vega/Glaucus gull	Novo Chaplino, Siberia	Si
43	Vega/Glaucus gull	Novo Chaplino, Siberia	CT4
44	Vega/Glaucus gull	Novo Chaplino, Siberia	CT3
45	Vega/Glaucus gull	Novo Chaplino, Siberia	CT4
46	Vega/Glaucus gull	Novo Chaplino, Siberia	CT6
47	Vega/Glaucus gull	Kolyuchin, Siberia	CT7
48	Vega/Glaucus gull	Kolyuchin, Siberia	CT2
49	Vega/Glaucus gull	Kolyuchin, Siberia	CT3
50	Vega/Glaucus gull	Kolyuchin, Siberia	CT7
51	Vega/Glaucus gull	Kolyuchin, Siberia	CT7
52	Vega/Glaucus gull	Kolyuchin, Siberia	CT7
53	Vega/Glaucus gull	Kolyuchin, Siberia	Si
54	Spoon-billed sandpiper	Kolyuchin, Siberia	CT3
55	Western sandpiper	Kolyuchin, Siberia	CT3
56	Western sandpiper	Kolyuchin, Siberia	CT3
57	Red-necked stint	Kolyuchin, Siberia	CT2
58	Red-necked phalarope	Kolyuchin, Siberia	CT3
59	Red-necked stint	Kolyuchin, Siberia	CT3
60	Emperor/Brent goose	Kolyuchin, Siberia	CT3
61	Emperor/Brent goose	Kolyuchin, Siberia	CT3
62	Emperor/Brent goose	Kolyuchin, Siberia	CT3
63	Emperor/Brent goose	Kolyuchin, Siberia	Si
64	Emperor/Brent goose	Kolyuchin, Siberia	CT3
65	Emperor/Brent goose	Kolyuchin, Siberia	CT3
66	Emperor/Brent goose	Kolyuchin, Siberia	CT3
67	Emperor/Brent goose	Kolyuchin, Siberia	CT3

68	Emperor/Brent goose	Kolyuchin, Siberia	CT3
69	Emperor/Brent goose	Kolyuchin, Siberia	CT3
70	Emperor/Brent goose	Kolyuchin, Siberia	CT3
71	Emperor/Brent goose	Kolyuchin, Siberia	CT3
72	Emperor/Brent goose	Kolyuchin, Siberia	CT2
73	Emperor/Brent goose	Kolyuchin, Siberia	CT3
74	Emperor/Brent goose	Kolyuchin, Siberia	CT3
75	Emperor/Brent goose	Kolyuchin, Siberia	CT5
76	Brent goose	Wrangel, Siberia	Si
77	Brent goose	Wrangel, Siberia	CT3
78	Vega/Glaucus gull	Wrangel, Siberia	CT8
79	Snow goose	Wrangel, Siberia	CT9
80	Snow goose	Wrangel, Siberia	Si
81	Snow goose	Wrangel, Siberia	CT9
82	Snow goose	Wrangel, Siberia	CT10
83	Snow goose	Wrangel, Siberia	CT10
84	Snow goose	Wrangel, Siberia	Si
85	Snow goose	Wrangel, Siberia	CT2
86	Pintail	Barrow, Alaska, USA	CT8
87	Pintail	Barrow, Alaska, USA	Si
88	Iceland/Glaucus gull	Thule, Greenland	Si
89	Iceland/Glaucus gull	Thule, Greenland	Si
90	Iceland/Glaucus gull	Thule, Greenland	CT11
91	Iceland/Glaucus gull	Thule, Greenland	CT11
92	Iceland/Glaucus gull	Thule, Greenland	CT2
93	Iceland/Glaucus gull	Thule, Greenland	Si
94	Iceland/Glaucus gull	Thule, Greenland	CT5
95	Iceland/Glaucus gull	Thule, Greenland	CT3
96	Iceland/Glaucus gull	Thule, Greenland	CT2
97	Iceland/Glaucus gull	Thule, Greenland	Si

*Western sandpiper, *Calidris maurii*; Dunlin, *Calidris alpina*; red-necked stint, *Calidris ruficollis*; yellow wagtail, *Motacilla flava*; Kittiwake, *Rissa tridactyla*; Vega/Glaucus gull, *Larus vegae/L. hyperboreus*; spoon-billed sandpiper, *Eurynorhychos pygmeus;* red-necked phalarope, *Phalaropus lobatus*; Emperor/Brent goose, *Anser canagica/Branta bernicla*; snow goose, *Anser caerulescens*; Pintail, *Anas acuta*; Iceland/Glaucus gull, *Larus glaucoides/Larus hyperboreus*.
†CT, common type; Si, single type.
